# Association between Sperm Morphology and Altered Sperm microRNA Expression

**DOI:** 10.3390/biology11111671

**Published:** 2022-11-17

**Authors:** Maja Tomic, Luka Bolha, Joze Pizem, Helena Ban-Frangez, Eda Vrtacnik-Bokal, Martin Stimpfel

**Affiliations:** 1 Department of Human Reproduction, Division of Obstetrics and Gynaecology, University Medical Centre Ljubljana, 1000 Ljubljana, Slovenia; 2 Institute of Pathology, Faculty of Medicine, University of Ljubljana, 1000 Ljubljana, Slovenia; 3 Faculty of Medicine, University of Ljubljana, 1000 Ljubljana, Slovenia

**Keywords:** sperm morphology, microRNA, teratozoospermia, male infertility

## Abstract

**Simple Summary:**

Sperm morphology is usually determined subjectively, and studies focused exclusively on sperm morphology are scarce. There is a global tendency to find objective markers of spermatozoa quality, including sperm morphology, as this will allow for a more personalized approach to managing and treating male infertility. MicroRNAs (miRNAs) are widely recognized as promising putative and objective biomarkers. In our study, we included 15 patients with normal sperm morphology and 13 patients with abnormal sperm morphology. We determined the expression profiles of 13 different miRNAs in the sperm of these participants and revealed that 9 miRNAs could serve as potential biomarkers of sperm morphology in spermatozoa.

**Abstract:**

Evaluation of male infertility has been based on semen analysis for years. As this method can be subjective at times, there is a scientific tendency to discover stable and quantifiable biomarkers. This study included 28 couples who underwent an in vitro fertilization/intracytoplasmic sperm injection (IVF/ICSI) cycle. The couples were assigned into two groups, according to sperm morphology. Couples where the males were normozoospermic were placed in the control group (15 participants), while couples where males had teratozoospermia were placed in the study group (13 participants). Thirteen candidate miRNAs were selected for qPCR analysis, based on our literature search. We determined significant under-expression of nine miRNAs (miR-10a-5p/-15b-5p/-26a-5p/-34b-3p/-122-5p/-125b-5p/-191-5p/-296-5p and let-7a-5p) in spermatozoa from patients with teratozoospermia compared to the controls, whereas expression levels of four miRNAs (miR-92a-3p/-93-3p/-99b-5p/-328-3p) did not significantly differ between the study and control groups. The expression levels of all 13 included miRNAs were significantly positively correlated with each other and significantly positively associated with spermatozoa morphology, excluding miR-99b-5p. There were no other significant associations between miRNA expression and sperm quality parameters. Only expression levels of miR-99b-5p were significantly positively correlated with good-quality day 3 embryo rate (*ρ* = 0.546; *p* = 0.003), while other variables of the IVF/ICSI cycle outcome showed no significant associations with miRNA expression profiles. This is one of the rare studies providing an insight directly into miRNA profiles in regard to sperm morphology. We identified nine miRNAs that could serve as biomarkers of spermatozoa quality in regard to teratozoospermia.

## 1. Introduction

The male factor of infertility is involved in up to a half of all cases of infertility [1,2]. The diagnosis of male infertility has been primarily based on semen analysis [2,3], which includes evaluation of sample volume and pH levels [3,4], as well as the number of spermatozoa and their concentration, motility and morphology. According to some accounts, these parameters are poor predictors of pregnancy, as their assessment is partially qualitative and partially based on the experience and subjective readings of the laboratory personnel performing the analyses [4]. Therefore, there are tendencies to discover and implement other, more suitable markers for semen quality analysis [3].

In recent years, altered microRNAs (miRNAs) in spermatozoa and seminal plasma have emerged as promising biomarkers for male infertility, and analyses of miRNA dysregulation have enabled more detailed insights into the biological processes of male infertility. Studies have revealed that sperm-derived miRNAs are differentially expressed in specific patterns, depending on the male infertility phenotype [2,5]. The majority of these studies have been conducted in regard to sperm concentration and motility [5,6], whereas data on the interrelation between miRNA dysregulation and sperm morphology remain scarce. Furthermore, a study by Salas-Huetos et al. discovered that miRNA expression profiles differ between spermatozoa from normozoospermic fertile and infertile males, which suggests that differential miRNA expression patterns could be used as biomarkers for infertility, even in the absence of the typical infertility phenotype [7].

Overall, environmental factors contribute a great deal to epigenetic alterations in spermatozoa, including miRNA expression [8]. Studies have shown that paternal obesity and dietary changes contribute crucially to miRNA dysregulation in spermatozoa, which may also further dictate offspring metabolism [9,10]. Similarly, alterations in miRNA expression, associated with decreased fertility in males, have been determined in sperm and seminal plasma from patients using prescription drugs and illegal substances, including verapamil [11] and heroin [12], respectively. In addition, a study by Jia et al. on animal models discovered miRNAs the expression profiles of which differed in relation to the age of the animals [13], which suggested that such interrelations could also apply in humans. Generally, these studies imply that miRNA dysregulation plays a notable role in male infertility; nevertheless, the impaired miRNA regulatory functions affecting spermatogenesis, spermatozoa motility and morphology remain poorly understood. Furthermore, several studies have discovered a positive correlation between sperm morphology and paternal age advancement [14,15,16].

Teratozoospermia is thought to indicate different structural defects or dysfunctions and has been regarded by some as one of the more valuable predictors of fertilization potential and pregnancy outcomes in conventional in vitro fertilization (IVF) [17,18,19]. This has been contradicted by several novel studies, which concluded that teratozoospermia has a limited predictive value for pregnancy outcome. In a study by Zhou et al., the results indicated that couples with teratozoospermia had significantly lower optimal embryo rates compared to couples with normal sperm morphology. This was only noted in IVF cycles, however, as the study did not observe the same results in cases where the intracytoplasmic sperm injection (ICSI) method was used [19]. 

The primary objective of this study was to assess the expression profiles of selected miRNAs in human sperm from patients with teratozoospermia and to determine whether the expression levels of these miRNAs are associated with IVF/ICSI cycle outcomes. With the inclusion of spermatozoa from 28 male patients, comprising 13 patients with teratozoospermia and 15 controls, we determined a distinct miRNA expression signature which seems to play a notable role in teratozoospermia pathophysiology and may eventually serve as a putative biomarker for male infertility. 

## 2. Material and Methods

### 2.1. Study Design

The current prospective study included 28 couples who were treated due to infertility at the Department of Human Reproduction, University Medical Centre (UMC) Ljubljana, Slovenia. Inclusion criteria for females was age <38 years and a good response to ovarian stimulation (with at least six oocytes retrieved), whereas there were no age or other restrictions for males. Twenty-five women underwent a short antagonist protocol, while three women underwent a long agonist protocol for ovarian hyperstimulation. Both protocols were conducted as described previously [20]. Male patients were invited to participate in the study based on their diagnostic spermiograms. To confirm sperm morphology, semen was reevaluated before its preparation for the IVF/ICSI procedure. We included sperm samples from a total of 28 male patients, of which 13 patients had teratozoospermia (the study group) and 15 patients had normozoospermia (the control group). Teratozoospermia was defined when less than 4% of the spermatozoa exhibited a normal morphology [21]. The included couples were divided into the two patient groups, according to the presence of abnormal sperm morphology in male patients. The study was approved by the National Medical Ethics Committee of the Republic of Slovenia (approval no. 0120-243/2019/6) and was performed in concordance with the Declaration of Helsinki. 

### 2.2. Semen Preparation

For the IVF/ICSI procedure, semen samples were prepared as previously described [22]. Briefly, the volumes of ejaculates were assessed using a graduated disposable pipette, whereas sperm concentration and total motility were assessed using 20 μm disposable counting slides with inbuilt 10 × 10 grids (CellVision). Sperm motility was evaluated under a phase contrast microscope (400× magnification), and spermatozoa were classified only as motile or immotile. Spermatozoa morphology was evaluated using pre-stained morphology slides (Cell-Vu), according to strict Tygerberg criteria. After initial assessment, the samples were prepared using density gradient centrifugation (DGC) (a 100% layer of Pure-Sperm 100 (Nidacon) and a 40% layer of PureSperm 100) for 20 min at 225× *g* at room temperature in order to remove immotile spermatozoa, somatic cells, cell debris and other impurities and to concentrate motile spermatozoa. Then, the 100% layer was washed in 4 mL of Sperm Preparation Medium (Origio), which was followed by centrifugation for 10 min at 300× *g* at room temperature. After centrifugation, the supernatant was discarded, and 0.3–1 mL of Sperm Preparation Medium was added to the pelleted spermatozoa cells to enable the swim-up of the most motile spermatozoa, which was performed in an incubator at 37 °C. After approximately 2 h of incubation, the samples were prepared either for ICSI or for conventional insemination of the cumulus–oocyte complexes (COCs). After the IVF/ICSI procedure, each remaining individual semen sample was resuspended in the medium and sperm concentration was reevaluated using disposable counting slides. If the number of spermatozoa was at least 5 × 10^6^, the sample was washed with PBS and in most cases stored in liquid nitrogen (*n* = 24) or at −80 °C, until the RNA isolation was performed. Sperm samples stored at −80 °C (*n* = 4) were used in an RNA isolation procedure within one week of preparation.

### 2.3. Embryo Culture and Embryo Transfer 

Embryo culture and embryo transfer were performed as previously described [22]. Briefly, normally fertilized oocytes (with two pronuclei) were cultured either in a continuous culture medium SAGE 1-Step (Origio) or in sequential G-series media (Vitrolife, Västra Frölunda, Sweden). In cases of sequential G-series media usage, the embryos were first cultured in G-1 Plus medium until the third day, when they were transferred to G-2 Plus medium (both obtained from Vitrolife). In cases where there were only one or two embryos, they were transferred into the uterus on the third day of development, at the cleavage stage. In most cases, there were >2 embryos, and embryo transfer was performed on day five, at the blastocyst stage. All embryo transfers were performed using a Guardia™ Access Embryo Transfer Catheter (Cook Medical, Bloomington, IN, USA), where, maximally, one or two embryos were transferred. Supernumerary embryos, upon reaching the blastocyst stage, were vitrified.

### 2.4. RNA Isolation

Total RNA was isolated from pelleted 5–40 × 10^6^ spermatozoa with an miRNeasy Mini Kit (217004, Qiagen, Germany), according to the manufacturer’s protocol, with a modified sample lysis step. Briefly, sperm samples were homogenized in 700 µL QIAzol Lysis Reagent (Qiagen) supplemented with β-mercaptoethanol (10 µL/mL) by 2 min rigorous vortexing. Cell homogenates were then lysed with 5 mm (mean diameter) stainless steel beads on the TissueLyser LT (69980, Qiagen, Germany) for 4 min at 50 Hz, followed by another 2 min of rigorous vortexing. Other isolation steps were performed according to the original protocol. The yield and purity of isolated RNA were assessed with a NanoDrop^TM^ One and with the Qubit^TM^ RNA HS Assay Kit (Q32855) on the Qubit^TM^ 3.0 Fluorometer (all obtained from Thermo Fisher Scientific, Waltham, MA, USA). All isolated total RNA samples were stored at −70 °C until they were used in reverse transcription and cDNA synthesis. Notably, no major discrepancies in overall yield and purity were observed between RNA samples of sperm stored in liquid nitrogen and sperm stored at −80 °C. To avoid multiple freeze–thaw cycles, all RNA samples were reverse transcribed at once in one series.

### 2.5. Reverse Transcription and Quantitative Real-Time PCR

Reverse transcription was performed with the miRCURY LNA RT Kit (339340, Qiagen, Germany) in 10 µL reaction volumes, as described previously [23]. Briefly, each reaction mixture contained 2 µL 5× miRCURY RT Reaction Buffer, RNase-free water, 1 µL 10× miRCURY RT Enzyme Mix, 0.5 µL UniSp6 RNA spike-in and 10 ng (1 ng/µL) total RNA template. 

Quantitative real-time PCR (qPCR) was performed in 10 µL reaction mixtures on the Rotor-Gene Q real-time PCR cycler (Qiagen, Germany). All qPCR analyses were performed in accordance with the Minimum Information for Publication of Quantitative Real-Time PCR Experiments (MIQE) guidelines [24] with a miRCURY SYBR Green PCR Kit (339347, Qiagen, Germany) and miRCURY LNA miRNA PCR Assays (339306; Qiagen, Germany), as previously described [23]. Briefly, each 10 µL qPCR reaction mixture contained 5 µL 2× miRCURY SYBR Green Master Mix, 1 µL PCR primer mix, 1 µL RNase-free water and 3 µL (0.05 ng) cDNA template. All qPCR reactions were performed in duplicate. The UniSp6 primer assay was used as a reverse transcription positive control and as an inter-plate calibrator. Thirteen candidate miRNAs were selected for qPCR analysis, based on their previously determined roles in spermatogenesis, fertilization and preimplantation embryonic development and on the association between their altered expression levels and sperm quality in patients with infertile ejaculates, characterized by a decreased number of spermatozoa and decreased motility [2,25,26,27,28,29,30,31,32,33]. The included miRNA primer assays are listed in Table S1.

In order to determine relative miRNA expression levels, a cDNA pool was generated, comprising cDNA samples from all included sperm samples (*n* = 28; c = 1 ng/µL). To determine the qPCR amplification efficiency (E) of each included miRNA primer assay, validation curves were generated from an eight-step, two-fold dilution series of the cDNA pool. All qPCR primer E reactions were performed in triplicate. Primer E values were calculated from the slopes of the validation curves, using *E* = 10^(−1/*slope of the standard curve)*^ [34], and used in subsequent calculations. Primer E of the endogenous reference (ER), used for data normalization, was obtained from the slope of the validation curve after geometrically averaging quantification cycle (Cq) values of SNORD38B, SNORD44 and SNORD49A reference primer assays for each validation curve dilution. The geometric means of multiple reference miRNA primer assays (SNORD38B, SNORD44 and SNORD49A) were used as ERs to enhance the accuracy of data normalization [35]. The E values determined for each miRNA primer assay and the characteristics of the validation curves are presented in Table S2. Before performing further calculations and data normalization, we used the determined primer E of each target miRNA and ER to efficiency-correct the exported Cq data of the qPCR analyses, as described previously [36]. The efficiency-corrected Cq values were then used for the calculation of miRNA expression levels by the 2^−ΔΔ*Ct*^ method [37], where ΔΔ*Ct* = ((*Cq_target miRNA_* − *Cq_ER_*)*_Study group_* − (*Cq_target miRNA_* − *Cq_ER_*)*_Control group_*). Efficiency-corrected *Cq* values of the ERs (geometric means of SNORD38B, SNORD44 and SNORD49A reference primer assay *Cq* values) were used for data normalization in all cases. The efficiency-corrected *Cq* values used for the data normalization and calculation of relative miRNA expression levels are presented in Table S3. 

### 2.6. Statistical Analysis

Statistical analysis was performed with the IBM SPSS Statistics 27.0 software (IBM Corporation, USA). Q–Q plots, Kolmogorov–Smirnov and Shapiro–Wilk tests were used to assess the normality of data distributions. Following a normal distribution, the unpaired Student’s *t*-test was used to evaluate differences between the relative miRNA expression levels and most of the IVF/ICSI cycle outcomes between the patient study and control groups. Sperm volume, concentration, total sperm count and motility were not distributed normally; therefore, we assessed differences in these factors between the groups using the Mann–Whitney *U* test. Where appropriate (in the comparison of proportions), Pearson’s chi-square test or Fisher’s exact test was applied. We also tested for correlations between different variables using Spearman’s correlation test. A *p*-value of <0.05 was considered statistically significant in all cases.

## 3. Results

### 3.1. Patient Characteristics

Twenty-eight couples who were treated due to infertility at the UMC Ljubljana were included in this prospective study, of which 13 were in the study group and 15 were in the control group. The results from andrological and embryological perspectives are presented in Table 1 and Table 2, respectively. Overall, sperm volume, concentration, total sperm count and sperm motility did not significantly differ between the study and control patient groups. We observed a significantly larger percentage of spermatozoa with abnormal morphology in the study group compared to the controls (*p* < 0.001), which coincided with our patient group formation based on sperm morphology (Table 1). Considering the factor of infertility, there were four cases of only male factor infertility (teratozoospermia) in the study group and zero cases in the control group. Further, there were 0 cases of only female infertility in the study group and 11 cases (1 tubal factor, 1 septum, while the other cases were combinations of endometriosis, endocrine, tubal and uterine factors) in the control group. When considering both factors of infertility, there were nine cases (five cases of endocrine factors, while the others were combinations of endometriosis, endocrine, tubal and uterine factors) in the study group and zero cases in the control group. The mean female and male ages, as well as body mass indexes (BMIs), were similar between the groups, and when we compared the outcomes of IVF/ICSI cycles in terms of oocytes, embryos and pregnancies the only significant difference we observed was a higher rate of degenerated oocytes in the study group (*p* = 0.040). All other outcomes from the mentioned perspectives were similar between the study and control patient groups, and are presented in Table 2.

### 3.2. MiRNA Expression in Spermatozoa 

**Figure 1:**
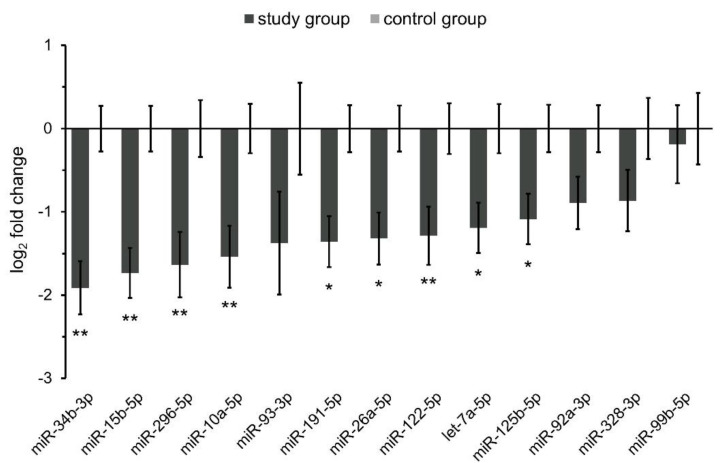
MicroRNA (miRNA) expression levels in spermatozoa from patients with teratozoospermia. Expression profiles of 13 selected miRNAs in spermatozoa from the study group (*n* = 13) compared to the controls (*n* = 15). The bars represent the means of log_2_ fold change values ± SDs. Data were evaluated using the unpaired Student’s *t*-test. An asterisk indicates significance with respect to the control group (* *p* < 0.05; ** *p* < 0.01). A *p*-value of < 0.05 was considered statistically significant.

Our analysis revealed that all of the 13 selected miRNAs were under-expressed in spermatozoa from the study group (13 patients with teratozoospermia), under our experimental conditions (Figure 1). As determined, miR-10a-5p/-15b-5p/-26a-5p/-34b-3p/-122-5p/-125b-5p/-191-5p/-296-5p and let-7a-5p were significantly under-expressed 3.8- to 2.1-fold (all *p* ≤ 0.034) when compared to the controls (Figure 1), while expression levels of the other four miRNAs, including miR-92a-3p/-93-3p/-99b-5p/-328-3p, did not significantly differ between patient groups.

### 3.3. Correlations between miRNA Expression Levels in Spermatozoa, Semen Parameters and IVF/ICSI Cycle Outcomes

We used Spearman’s correlation test to evaluate associations between the miRNA expression levels in spermatozoa and the basic parameters of semen quality and selected variables of the IVF/ICSI cycle outcome (Table 3, Table 4 and Table S4). Overall, we revealed a strong significant positive correlation between the expression levels of all tested miRNAs (all *ρ* > 0.528; *p* < 0.001) (Table S4). Moreover, sperm morphology was significantly positively correlated with the expression of all the tested miRNAs, except miR-99b-5p. There were no other significant associations between miRNA expression and sperm quality parameters (Table 4). When miRNA expression was correlated with selected variables of the IVF/ICSI cycle outcome, only expression levels of miR-99b-5p were significantly positively correlated with good-quality day 3 embryo rate (*ρ* = 0.546; *p* = 0.003). Other variables of the IVF/ICSI cycle outcome showed no significant associations with miRNA expression profiles (Table 4).

When assessing associations between selected variables of the IVF/ICSI cycle outcome and basic parameters of semen quality, we determined that the number of blastocysts was significantly positively correlated with the total number of embryos (*ρ* = 0.716; *p* < 0.001). In addition, the number of blastocysts was significantly positively correlated with the number of good-quality day 3 embryos (*ρ* = 0.565; *p* = 0.002), the number of good-quality blastocysts (*ρ* = 0.880; *p* < 0.001), sperm concentration (*ρ* = 0.437; *p* = 0.020) and total sperm count (*ρ* = 435; *p* = 0.021) (Table 3). Similarly, the number of good-quality blastocysts was significantly positively correlated with the total number of embryos (*ρ* = 0.705; *p* < 0.001), the number of good-quality day 3 embryos (*ρ* = 0.579; *p* = 0.002), a good-quality blastocyst rate (*ρ* = 0.609; *p* = 0.001), sperm concentration (*ρ* = 0.507; *p* = 0.006) and total sperm count (*ρ* = 0.395; *p* = 0.038). In addition, the number of good-quality day 3 embryos was significantly positively correlated with the number of oocytes (*ρ* = 0.466; *p* = 0.014), the number of embryos (*ρ* = 0.667; *p* < 0.001) and a good-quality day 3 embryo rate (*ρ* = 0.613; *p* = 0.001) (Table 3). Significant associations were also determined between the numbers of oocytes and embryos (*ρ* = 0.647; *p* < 0.001) and between male age and good-quality blastocyst rate (*ρ* = 0.379; *p* = 0.047). Total sperm count was significantly associated with sperm volume and concentration (both *ρ* > 0.408; *p* < 0.031) (Table 3).

## 4. Discussion

Our study of miRNA expression in spermatozoa revealed a distinct spermatozoa-phenotype-related miRNA expression profile in patients with teratozoospermia compared to men with normozoospermia and emphasized the potential for using altered miRNA signatures as putative non-invasive biomarkers for male infertility. The miRNA dysregulation in the sperm of men with teratozoospermia has not been widely researched. Our results therefore contribute an additional insight into the complexity of the molecular mechanisms underlying male infertility through alterations in spermatozoa morphology.

There have been several efforts to implement and optimize miRNA-based biomarker panels for the diagnosis of male infertility. Corral-Vazquez et al. published a study in 2019 [2], in which they tried to find and optimize an optimal panel of biomarkers for male infertility using stable miRNA pairs based on the correlated expression patterns. Among the many miRNAs thought to be relevant to teratozoospermia, five were the same as those included in our research: miR-296-5p, miR-328-3p, miR-92a-3p, miR-125b-5p and miR-99b-5p. Since their study was focused on miRNA pairs, the pair miR-296-5p/miR-328-3p stands out, as both miRNAs were prominent in our research as well. They determined that the pair was expressed in high percentages not only in cases of teratozoospermia but also in asthenozoospermia, oligozoospermia and, somewhat surprisingly, in normozoospermia. Unfortunately, Corral-Vazquez et al.could not prove the differences in the aforementioned miRNA pair between fertile and infertile patients in the validation stage. There were multiple other miRNAs that were not exclusive to teratozoospermia but also present in other seminal alterations [2]. In our study, we determined a positive correlation between expression levels of miR-296-5p and miR-328-3p; however, only the expression of miR-296-5p was significantly under-expressed in teratozoospermia cases compared to control subjects with normozoospermia. Furthermore, our results indicated a possible connection between miR-296-5p expression and successful pregnancy, but the results did not reach the threshold of statistical significance. Based on the results obtained by Corral-Vazquez et al., miR-296-5p is a poor indicator of teratozoospermia, as it lacks sensitivity and specificity. In our study, we determined the expression of miR-296-5p as well and revealed that miR-296-5p expression levels correlated with sperm morphology. A study by Salas-Huetos et al. in 2015 assessed 734 different miRNAs in human spermatozoa using qPCR. They identified miR-15b-5p and miR-34b-3p as possible markers in men with asthenozoospermia and oligozoospermia but not in men with teratozoospermia [5].

In a later study, Salas-Huetos et al. compared normozoospermic sperm of fertile and infertile men and identified 57 differentially expressed miRNAs [7]. Of these, four miRNAs showed a positive correlation with spermatozoa phenotype in our study as well, including let-7a-5p, miR-93-3p, miR-15b-5p and miR-191-5p (Table 4). Notably, Gholami et al. (2021) identified miR-182-5p, miR-192-5p, miR-204-5p and miR-493-5p as part of a regulatory network of cystein-rich secretory protein 3 (CRISP3) isoforms in spermatozoa from men with teratozoospermia and proposed overexpressed miR-182-5p, miR-192-5p and miR-493-5p as putative biomarkers of teratozoospermia [38]. These studies, together with our study, show that, while a correlation between sperm morphology and sperm miRNA in all likelihood exists, there are still not very many data related to the influence of sperm miRNA expression on IVF/ICSI cycle outcome. Considering only morphology, it was suggested that teratozoospermia has a low predictive value for IVF/ICSI cycle outcome [19]. It was also suggested that isolated teratozoospermia might not influence IVF/ICSI outcome [39]. A study on animal models, for example, indicated that fertilization of oocytes with morphologically abnormal spermatozoa (e.g., grossly misshapen heads) can lead to normal embryonic development and ultimately to the birth of healthy offspring [40]. Association analysis of miRNA expression and IVF/ICSI cycle outcome in our study yielded only one significant correlation, which was a significant positive correlation between expression levels of miR-99b-5p and good-quality day 3 embryo rate. This miRNA has been previously associated with IVF/ICSI cycle outcome, its expression having been found to be down-regulated in culture media of blastocysts achieving pregnancy compared to failed implantation cases [41]. Conversely, miR-99b-5p was up-regulated in the endometrial fluid of an implantative group of patients [42]. These opposing data suggest that the same miRNAs might be crucial to achieve successful pregnancy, although their expression patterns differ in each stage of reproduction. The importance of miRNAs and other small RNAs in achieving successful pregnancy and live birth has been explored in detail in mouse models as well. For instance, Conine et al. discovered in their study that paternally transmitted small RNAs are crucial for achieving live offspring. Furthermore, they discovered that these small RNAs are present in spermatozoa after maturation in the cauda epididymis while being absent from spermatozoa derived from the caput epididymis. While their study showed that caput-derived spermatozoa were capable of fertilization and even blastocyst formation, they did not lead to live birth of offspring. Interestingly, if caput-derived zygotes were injected with epididymosomal-derived small RNAs, this rescued the preimplantation molecular profile and even led to live birth of offspring [43].

A limited number of participants represents the main limitation of the current study. By expanding the number of participants, we could obtain additional crucial data on the assessed parameters which could support a more reliable statistical evaluation and reveal associations that currently remain concealed. Another limitation in correlating miRNA expression profiles with IVF/ICSI outcomes could be the method of choice for oocyte fertilization, because in the study group mostly ICSI was used. In addition, among the biggest limitations of this and other studies assessing the interrelations between miRNA function and/or expression and spermatozoa abnormalities is a significant lack of comprehensive integrative approaches, which would enable the elucidation of only the key miRNAs involved in infertility, instead of a large variety of miRNAs. Implementation of such studies would undoubtedly result in the identification of more reliable putative biomarkers for male and female infertility amongst many possible targets. Furthermore, environmental factors, including patient age, obesity, dietary changes and prescription and illegal drug usage, should be considered in patients when assessing miRNA dysregulation in spermatozoa and seminal plasma, due to their notable influences on epigenetics. Finally, functional studies on miRNA dysregulation in the male reproductive system, including sperm and seminal plasma, might reveal miRNA-dependent mechanisms involved in teratozoospermia and other conditions underlying male infertility and aid in identifying novel biomarkers.

## 5. Conclusions

We have revealed a distinct miRNA expression profile in men with teratozoospermia which indicates the potential role of miRNA dysregulation in male infertility. Since we focused our research solely on teratozoospermia and its relation to normozoospermia, our results represent a notable scientific contribution. We discovered a significant correlation between miR-99b-5p and good-quality day 3 embryo rate. Nevertheless, further in-depth studies are needed, with the enrolment of more patients and consideration of the most prevalent environmental factors among participants, to identify key miRNAs and other molecular markers underlying male infertility.

## Figures and Tables

**Table 1:** Baseline semen parameters of the samples used in the IVF/ICSI cycles.

Parameter	Study Group	Control Group	*p*-Value
Sperm volume (ml)	2.0 (2.0–3.0)	2.0 (1.5–3.0)	0.786
Sperm concentration (×10^6^/mL)	60 (45–90)	80 (70–100)	0.088
Total sperm count (×10^6^)	150 (90–200)	200 (150–240)	0.058
Total motility (%)	60 (55–70)	70 (60–70)	0.294
Sperm morphology (%)	2.0 (1.2–2.5)	10 (9.0–14.4)	<0.001 ***

Values reported as medians with interquartile ranges (Q1–Q3). An asterisk indicates significance with respect to the control group (*** *p* < 0.001). A *p*-value of < 0.05 was considered statistically significant.

**Table 2:** Outcomes of the IVF/ICSI cycles in terms of oocytes, embryos and pregnancies.

Parameter	Study Group	Control Group	*p*-Value
Number of cycles	13	15	
Female age (years) (mean ± SD)	32.5 ± 3.3	32.9 ± 3.9	0.764
Male age (years) (mean ± SD)	33.9 ± 3.8	36.1 ± 4.8	0.194
Gonadotrophins used in IE (mean ± SD)	1942 ± 699	1985 ± 674	0.871
Only male factor of infertility (teratozoospermia)	4	0	
Only female factor of infertility	0	11	
Male and female factor infertility	9	0	
Female BMI	25.6 ± 6.4	22.8 ± 5.1	0.185
Male BMI	27.2 ± 4.8	25.7 ± 4.3	0.345
Total number of retrieved oocytes (mean per cycle ± SD)	166 (12.8 ± 5.5)	185 (12.3 ± 4.0)	0.811
Normally fertilized oocytes per number of retrieved oocytes; *n* (rate (%))	88/166 (53.0%)	114/185 (61.6%)	0.104
Immature oocytes; *n* (rate (%))	24 (14.5%)	32 (17.3%)	0.467
Degenerated oocytes per number of retrieved oocytes; *n* (rate (%))	19/166 (11.4%)	10/185 (5.4%)	0.040 *
Polyploidies per number of retrieved oocytes; *n* (rate (%))	4/166 (2.4%)	8/185 (4.3%)	0.325
Cleaved embryos; *n* (% per zygotes)	86 (97.7%)	114 (100%)	0.189
Number of embryos per cycle (mean ± SD)	6.6 ± 4.6	7.6 ± 4.0	0.551
Number of embryos cultured until day 5/6	84	113	
Blastocysts per embryos cultured until day 5/6; *n* (rate (%))	39/84 (46.4%)	63/113 (55.8%)	0.195
Embryo utilization (transferred + frozen embryos); *n* (rate (%))	42 (48.8%)	64 (56.1%)	0.306
Number of frozen blastocysts (mean ± SD)	2.2 ± 3.4	3.3 ± 2.7	0.343
Cycles with at least one blastocyst; *n* (%)	11 (84.6%)	14 (93.3%)	0.583
Cryopreserved embryos; *n* (rate (% of all embryos))	29 (33.7%)	50 (43.9%)	0.146
Cycles with embryo cryopreservation; *n* (%)	9 (69.2%)	12 (80.0%)	0.670
Cycles with freezing without ET; *n* (%)	2 (15.4%)	1 (6.7%)	1
Cycles without freezing/without ET; *n* (%)	0 (0%)	0 (0%)	NA
Total number of fresh ETs	11	14	
Number of transferred embryos (mean ± SD)	1.2 ± 0.4	1.0 ± 0.0	0.467
Pregnancies; *n* (% per ET)	5 (45.5%)	6 (42.9%)	1
Pregnancies per oocyte aspiration (%)	38.5%	40.0%	1

IE, international unit; BMI, body mass index; ET, embryo transfer; NA, not applicable. An asterisk indicates significance with respect to the control group (* *p* < 0.05). A *p*-value of < 0.05 was considered statistically significant.

**Table 3:** Spearman’s correlation matrix of associations between selected variables of the IVF/ICSI cycle outcome and basic parameters of semen quality.

	Oocytes (*n*)	Embryos (*n*)	Blastocysts (*n*)	Good-Quality Day 3 Embryos (*n*)	Good-Quality Day 3 Embryo Rate	Good-Quality Blastocysts (*n*)	Good-Quality Blastocyst Rate	Pregnancy	Sperm Volume	Sperm Concentration	Sperm Motility	Sperm Morphology	Male Age	Total Sperm Count	Male BMI	Female BMI
Oocytes (*n*)	1															
Embryos (*n*)	0.647 ***	1														
Blastocysts (*n*)	0.338	0.716 ***	1													
Good-quality day 3 embryos (*n*)	0.466 *	0.667 ***	0.565 **	1												
Good-quality day 3 embryo rate	0.097	0.072	0.241	0.613 **	1											
Good-quality blastocysts (*n*)	0.234	0.705 ***	0.880 ***	0.579 **	0.172	1										
Good-quality blastocyst rate	−0.283	0.182	0.311	0.195	−0.121	0.609 **	1									
Pregnancy	0.219	0.086	0.238	0.254	0.182	0.110	−0.162	1								
Sperm volume	−0.034	−0.076	−0.001	−0.277	−0.123	−0.119	0.018	−0.151	1							
Sperm concentration	−0.004	0.182	0.437 *	0.360	0.248	0.507 **	0.269	0.170	−0.302	1						
Sperm motility	0.106	−0.066	0.011	−0.054	0.011	−0.035	−0.104	0.146	0.159	0.198	1					
Sperm morphology	−0.008	0.143	0.151	−0.052	−0.186	0.136	−0.026	0.027	0.081	0.181	0.183	1				
Male age	−0.189	0.031	−0.011	−0.104	−0.178	0.160	0.379 *	−0.231	0.142	−0.010	0.061	0.179	1			
Total sperm count	−0.024	0.101	0.435 *	0.063	0.151	0.395 *	0.271	−0.018	0.408 *	0.686 ***	0.266	0.278	0.077	1		
Male BMI	−0.309	−0.111	0.043	−0.098	0.084	−0.010	0.099	0.073	−0.133	−0.037	−0.266	−0.399	−0.095	−0.133	1	
Female BMI	−0.260	−0.252	−0.184	−0.150	0.0001	−0.211	−0.053	−0.217	−0.017	−0.304	−0.033	−0.330	−0.240	−0.358	0.677 **	1

Spearman’s correlation coefficients (*ρ*) between evaluated parameters are presented. A *p*-value of < 0.05 was considered statistically significant (* *p* < 0.05; ** *p* < 0.01; *** *p* < 0.001).

**Table 4:** Spearman’s correlation matrix of associations between selected variables of the IVF/ICSI cycle outcome, basic parameters of semen quality and miRNA expression levels in spermatozoa.

	miR-10a-5p	miR-15b-5p	miR-26a-5p	miR-34b-3p	miR-92a-3p	miR-93-3p	miR-99b-5p	miR-122-5p	miR-125b-5p	miR-191-5p	miR-296-5p	miR-328-3p
Oocytes (*n*)	−0.055	−0.083	0.021	−0.134	−0.005	0.070	0.012	−0.123	−0.092	−0.125	−0.108	−0.262
Embryos (*n*)	0.162	0.125	0.065	0.053	0.157	0.218	0.136	0.017	0.070	0.024	0.056	−0.176
Blastocysts (*n*)	0.191	0.197	0.129	0.160	0.080	0.110	0.028	0.057	0.056	0.065	0.233	−0.145
Good-quality day 3 embryos (*n*)	0.165	0.166	0.172	0.082	0.117	0.252	0.288	0.060	0.155	0.090	0.161	0.005
Good-quality day 3 embryo rate	0.230	0.198	0.337	0.199	0.272	0.225	0.546 **	0.187	0.316	0.225	0.337	−0.012
Good-quality blastocysts (*n*)	0.166	0.183	0.043	0.163	0.102	0.145	0.076	0.090	0.061	0.118	0.099	−0.036
Good-quality blastocyst rate	0.054	0.062	−0.061	0.032	−0.042	0.068	−0.032	0.037	−0.023	0.052	−0.161	0.298
Pregnancy	0.195	0.177	0.222	0.222	0.167	0.158	0.059	0.104	0.131	0.186	0.330	0.159
Sperm volume	0.039	0.044	0.157	0.045	0.066	−0.026	−0.114	0.135	0.067	0.021	0.094	0.179
Sperm concentration	0.038	0.047	−0.007	0.152	−0.096	−0.139	−0.110	0.036	−0.018	0.098	0.151	−0.238
Sperm motility	0.088	0.045	0.014	0.007	0.076	−0.023	−0.019	0.036	−0.057	0.031	0.073	0.094
Sperm morphology	0.540 **	0.568 **	0.403 *	0.540 **	0.400 *	0.453 *	0.090	0.454 *	0.453 *	0.522 **	0.532 **	0.462 *
Male age	0.005	−0.017	−0.047	−0.049	0.008	−0.071	−0.067	0.063	−0.044	−0.066	−0.134	−0.057
Total sperm count	0.071	0.072	0.136	0.183	−0.063	−0.103	−0.144	0.130	0.025	0.107	0.188	−0.134
Male BMI	0.012	−0.033	0-.032	0.045	0.064	−0.028	0.142	0.042	0.073	−0.023	0.039	−0.031

Spearman’s correlation coefficients (*ρ*) between evaluated parameters are presented. A *p*-value of < 0.05 was considered statistically significant (* *p* < 0.05; ** *p* < 0.01).

## Data Availability

Not applicable.

## Supplementary Materials

The following supporting information can be downloaded at: https://www.mdpi.com/article/10.3390/biology11111671/s1, Table S1: miRNA primer assays; Table S2: Characteristics of validation curves used for relative quantification of miRNA expression; Table S3: Quantification cycle (Cq) values used for data normalization and calculation of relative miRNA expression; Table S4: Spearman’s correlation matrix of associations between miRNA expression levels in spermatozoa from patients with teratozoospermia.
