# Supplementary material for: Association between Sperm Morphology and Altered Sperm microRNA Expression
Source: Biology (Basel). 2022 Nov 17;11(11):1671. doi: 10.3390/biology11111671 (PMC9687816; doi:10.3390/biology11111671)
Supplement: Supplementary file 1 [file biology-11-01671-s001.zip › biology-1980803-supplementary.pdf]

Supplementary Material

Table S1. miRNA primer assays.

**miRNA**	**Catalogue number**[a]
let-7a-5p	YP00205727
miR-10a-5p	YP00204778
miR-15b-5p	YP00204243
miR-26a-5p	YP00206023
miR-34b-3p	YP00204005
miR-92a-3p	YP00204258
miR-93-3p	YP00204470
miR-99b-5p	YP00205983
miR-122-5p	YP00205664
miR-125b-5p	YP00205713
miR-191-5p	YP00204306
miR-296-5p	YP00204436
miR-328-3p	YP00204364
SNORD38B[b]	YP00203901
SNORD44[b]	YP00203902
SNORD49A[b]	YP00203904
UniSp6[c]	YP00203954

[a] miRCURY LNA miRNA PCR Assay catalogue number (product number 339306, Qiagen, Germany).

[b] Reference miRNA PCR assays used for data normalization.

[c] miRNA PCR assay specific for amplification of UniSp6 RNA spike-in control.

Table S2. Characteristics of validation curves used for relative quantification of miRNA expression.

miRNA	Slope[a]	E[b]	$R^{2,c}$
**let-7a-5p**	–3.35	1.99	0.997
**miR-10a-5p**	–4.41	1.69	0.997
**miR-15b-5p**	–3.37	1.98	0.994
**miR-26a-5p**	–3.34	1.99	0.997
**miR-34b-3p**	–3.36	1.99	0.995
**miR-92a-3p**	–3.72	1.86	0.984
**miR-93-3p**	–3.38	1.98	0.984
**miR-99b-5p**	–3.35	1.99	0.984
**miR-122-5p**	–3.46	1.94	0.990
**miR-125b-5p**	–3.61	1.89	0.994
**miR-191-5p**	–3.57	1.90	0.988
**miR-296-5p**	–3.35	1.99	0.981
**miR-328-3p**	–3.88	1.81	0.983
**ER[d]**	–3.41	1.97	0.995

[a]Slope of the validation curve.

[b]qPCR miRNA primer assay amplification efficiency.

[c]Coefficient of determination of the validation curve.

[d]Endogenous reference, obtained by geometrically averaging Cq values of SNORD38B, SNORD44 and SNORD49A reference miRNA primer assays.

Table S3. Quantification cycle (Cq) values used for data normalization and calculation of relative miRNA expression.

	Study group													Control group														
**miRNA**	**sample-1**	**sample-2**	**sample-3**	**sample-4**	**sample-5**	**sample-6**	**sample-7**	**sample-8**	**sample-9**	**sample-10**	**sample-11**	**sample-12**	**sample-13**	**Ctrl-1**	**Ctrl-2**	**Ctrl-3**	**Ctrl-4**	**Ctrl-5**	**Ctrl-6**	**Ctrl-7**	**Ctrl-8**	**Ctrl-9**	**Ctrl-10**	**Ctrl-11**	**Ctrl-12**	**Ctrl-13**	**Ctrl-14**	**Ctrl-15**
**let-7a-5p[a]**	24.69	21.21	21.34	21.67	23.01	22.07	21.69	22.34	25.31	22.98	21.40	22.37	23.36	21.56	21.40	21.11	21.44	22.33	23.55	21.14	22.06	22.98	21.25	21.75	23.32	20.92	22.04	21.50
**miR-10a-5p[a]**	22.29	19.77	19.39	19.94	20.41	20.21	19.41	21.58	22.59	19.86	19.69	20.37	22.09	19.36	18.84	18.13	19.08	20.17	21.03	18.72	19.83	20.37	19.60	20.21	20.89	18.61	19.54	18.97
**miR-15b-5p[a]**	22.98	20.88	20.45	20.97	21.23	21.04	20.62	23.36	24.22	22.02	19.96	21.28	23.00	20.04	19.47	19.53	20.50	21.09	21.54	19.24	20.45	21.57	20.18	20.95	21.98	20.52	20.33	19.67
**miR-26a-5p[a]**	25.72	22.55	22.23	23.25	23.13	22.84	23.06	23.57	26.38	24.13	21.35	23.45	24.53	21.58	21.67	21.62	22.99	23.05	24.10	22.03	23.03	24.31	22.27	22.42	24.24	22.47	22.96	22.43
**miR-34b-3p[a]**	23.27	21.63	21.42	22.01	22.27	21.73	21.31	25.08	25.63	23.00	20.56	22.48	24.17	20.51	20.20	20.03	21.13	22.08	22.80	20.29	21.86	22.79	20.99	21.36	22.48	20.53	21.25	20.54
**miR-92a-3p[a]**	21.35	20.47	20.40	20.39	21.52	20.29	21.06	22.27	24.10	21.20	19.96	20.82	22.06	20.57	20.48	20.18	20.35	20.91	22.13	19.81	20.67	21.29	20.25	21.10	22.28	20.22	21.20	21.21
**miR-93-3p[a]**	31.21	29.25	29.68	29.02	29.54	28.98	29.70	NA	30.47	30.71	28.19	29.78	NA	29.51	28.37	26.98	29.05	30.17	29.86	27.88	28.16	30.64	29.19	29.66	30.05	29.29	29.32	28.78
**miR-99b-5p[a]**	30.82	26.90	27.89	27.91	28.89	27.01	28.63	28.62	31.30	28.96	29.08	29.77	30.64	28.09	28.87	27.88	28.69	28.82	29.83	28.62	29.35	29.39	28.63	30.51	NA	29.84	28.96	29.03
**miR-122-5p[a]**	25.27	23.71	23.18	23.39	23.67	23.86	23.81	26.07	26.78	24.83	22.74	24.23	25.92	22.91	22.90	22.02	23.84	24.51	24.87	23.01	23.97	24.92	22.80	23.08	25.78	23.09	24.04	22.91
**miR-125b-5p[a]**	23.21	21.30	20.44	21.22	21.74	20.74	21.06	23.49	24.65	21.73	20.99	21.65	23.41	20.50	20.68	20.56	21.44	21.58	22.45	20.87	21.79	22.59	21.39	21.48	22.83	20.12	21.67	20.97
**miR-191-5p[a]**	22.45	20.64	19.55	20.42	20.74	19.88	19.52	23.25	24.39	21.27	19.79	22.19	23.26	19.70	19.50	19.43	20.28	20.99	21.92	19.64	21.34	21.57	20.14	21.30	21.40	19.73	20.80	19.61
**miR-296-5p[a]**	29.85	26.32	26.27	26.31	27.70	27.15	26.66	28.95	31.49	26.78	26.05	26.65	29.00	26.06	24.81	25.71	25.85	27.33	28.61	25.21	26.72	28.08	26.08	26.41	28.63	25.36	26.56	26.14
**miR-328-3p[a]**	NA	23.65	NA	NA	23.74	NA	22.80	25.40	25.79	NA	22.81	23.51	NA	24.13	23.05	23.14	23.14	24.02	25.05	22.59	23.23	24.92	22.83	24.72	25.81	23.31	23.82	NA
**SNORD38B (ref.)[b]**	28.72	24.65	22.98	23.58	25.82	26.05	24.15	27.44	NA	26.85	25.56	25.80	29.70	25.10	25.14	26.90	25.97	26.77	29.71	27.66	26.23	27.59	25.22	26.23	30.33	27.19	28.38	25.00
**SNORD44 (ref.)[b]**	30.20	26.31	23.96	24.58	26.94	28.20	25.16	28.09	30.72	30.08	26.54	26.86	30.08	25.88	26.96	27.10	27.73	26.92	29.98	29.82	28.06	27.70	26.25	27.46	31.23	29.36	29.51	26.12
**SNORD49A (ref.)[b]**	NA	25.44	24.13	23.94	26.57	27.23	25.54	27.89	31.51	29.11	26.58	25.96	29.10	25.69	26.97	27.32	28.44	27.68	28.70	28.96	27.44	27.50	25.29	26.77	31.43	27.81	29.25	25.36
**ER[c]**	28.72	24.83	23.10	23.43	25.78	26.47	24.33	27.12	30.34	27.94	25.57	25.55	28.89	24.92	25.69	26.44	26.68	26.45	28.73	28.09	26.56	26.91	24.95	26.15	30.22	27.41	28.32	24.86

[a]Inter-plate-calibrated and efficiency-corrected target miRNA Cq values.

[b]Inter-plate-calibrated reference miRNA Cq values.

[c]Endogenous reference. Efficiency-corrected geometric mean of reference miRNA inter-plate-calibrated Cq values (SNORD38B, SNORD44 and SNORD49A), which was used for data normalization.

Table S4. Spearman's correlation matrix of associations between miRNA expression levels in spermatozoa from patients with teratozoospermia.

	miR-10a-5p	miR-15b-5p	miR-26a-5p	miR-34b-3p	miR-92a-3p	miR-93-3p	miR-99b-5p	miR-122-5p	miR-125b-5p	miR-191-5p	miR-296-5p	miR-328-3p
**miR-10a-5p**	1											
**miR-15b-5p**	0.965**	1										
**miR-26a-5p**	0.892**	0.901**	1									
**miR-34b-3p**	0.904**	0.950**	0.900**	1								
**miR-92a-3p**	0.904**	0.881**	0.826**	0.862**	1							
**miR-93-3p**	0.917**	0.946**	0.886**	0.884**	0.893**	1						
**miR-99b-5p**	0.714**	0.665**	0.662**	0.582**	0.758**	0.757**	1					
**miR-122-5p**	0.905**	0.918**	0.898**	0.938**	0.882**	0.915**	0.626**	1				
**miR-125b-5p**	0.945**	0.933**	0.889**	0.921**	0.924**	0.902**	0.755**	0.924**	1			
**miR-191-5p**	0.930**	0.937**	0.859**	0.941**	0.879**	0.871**	0.725**	0.895**	0.951**	1		
**miR-296-5p**	0.875**	0.899**	0.874**	0.913**	0.832**	0.784**	0.528**	0.840**	0.874**	0.840**	1	
**miR-328-3p**	0.852**	0.861**	0.781**	0.779**	0.881**	0.910**	0.636**	0.800**	0.844**	0.829**	0.731**	1

Spearman's correlation coefficients (ρ) between individual miRNA expression levels are presented. A p-value of < 0.05 was considered statistically significant (**$p < 0.001$).
